# School Meals as a Strategy to Prevent Childhood Obesity and Advance Food Equity: A Narrative Review

**DOI:** 10.1007/s13679-026-00697-5

**Published:** 2026-03-06

**Authors:** Gabriella M. McLoughlin, Juliana F. Cohen

**Affiliations:** 1 Department of Social and Behavioral Sciences, Barnett College of Public Health, Temple University, Philadelphia, United States; 2 Department of Nutrition and Public Health, Merrimack College, 315 Turnpike Street, North Andover, MA 01845 USA; 3 Department of Nutrition, Harvard T.H. Chan School of Public Health, 677 Huntington Ave, Boston, MA 02115 USA

**Keywords:** Universal School Meals, Childhood Obesity, Food Insecurity, Health Equity, School Nutrition Policy

## Abstract

**Purpose of Review:**

Childhood obesity and food insecurity are coexisting public health crises that disproportionately affect low-income and marginalized populations. Addressing these issues requires sustainable interventions and policies that improve nutritional access and equity. We therefore sought to synthesize evidence on the role of school meal programs, particularly Universal School Meals (USM), in preventing childhood obesity and advancing food security and nutrition equity through a narrative review.

**Recent Findings:**

School meals are among the healthiest food sources for US children and improve diet quality compared to meals from other sources. Policies including the Community Eligibility Provision increase participation, reduce stigma, and alleviate food insecurity. Evidence suggests USM adoption is associated with lower obesity prevalence and improved household financial stability, positioning school meals as a powerful equity-focused intervention.

**Summary:**

School meals represent a critical lever for reducing diet-related and obesity disparities and advancing food equity for children. Further research should explore implementation strategies and long-term outcomes to maximize health and social benefits.

## Introduction

Across the United States, childhood obesity is on the rise, and projected to increase exponentially so that by 2050, 43 million children and adolescents will have overweight or obesity [1]. Compared to high-income families, children and adolescents in low and middle-income families are more at risk for obesity, with those from the highest poverty households at greatest risk [2]. Further, risk for food insecurity is heavily linked to risk for obesity [3], and this relationship is heightened in economically marginalized populations [3]. For example, children living in low-income households and/or who identify as a racial or ethnic minority are more than twice as likely to experience food insecurity than their white, middle class counterparts [4]. Therefore, nutrition intervention and policies that address both food security and obesity among the highest risk populations are critical and intervention development should prioritize historically underserved populations to address growing inequities.

Universal School Meals (USM) is an innovative and overlooked policy intervention that has strong potential to mitigate food insecurity risk among US school-aged children. For the last six decades, the National School Lunch Program (NSLP) [5] and the School Breakfast Program [6] (SBP) have been the primary federal food safety net programs for school-aged children and have played a major role in combatting food insecurity among children from low-income households. As a policy, USM allows schools and districts to provide breakfast and lunch to all students for no cost if the proportion of eligible students (i.e., those whose families receive federal assistance or identify as low-income) is above 25% of school enrollment. Schools and districts are provided with financial reimbursement for the meals provided to students, thus greater participation provides financial stability through higher reimbursement amounts. Accordingly, the potential impact on hunger and food insecurity is significant. Over the past decade, there have been several policies to help expand USM to students at the school and district-level, and there is a growing movement to implement state-level USM policies to address growing inequalities in food security [7].

Given the growing focus on school meals nationwide, a closer look at the evidence behind USM and its potential as a key policy tool for preventing child obesity is warranted. Accordingly, the purpose of this narrative review is to provide an overview of the research conducted to examine USM as a potential strategy for preventing childhood overweight and obesity and promoting food equity. We will highlight the strengths and limitations of the evidence gathered thus far and provide recommendations for future research, action, and advocacy.

## Conceptual Framework

**Fig. 1:**
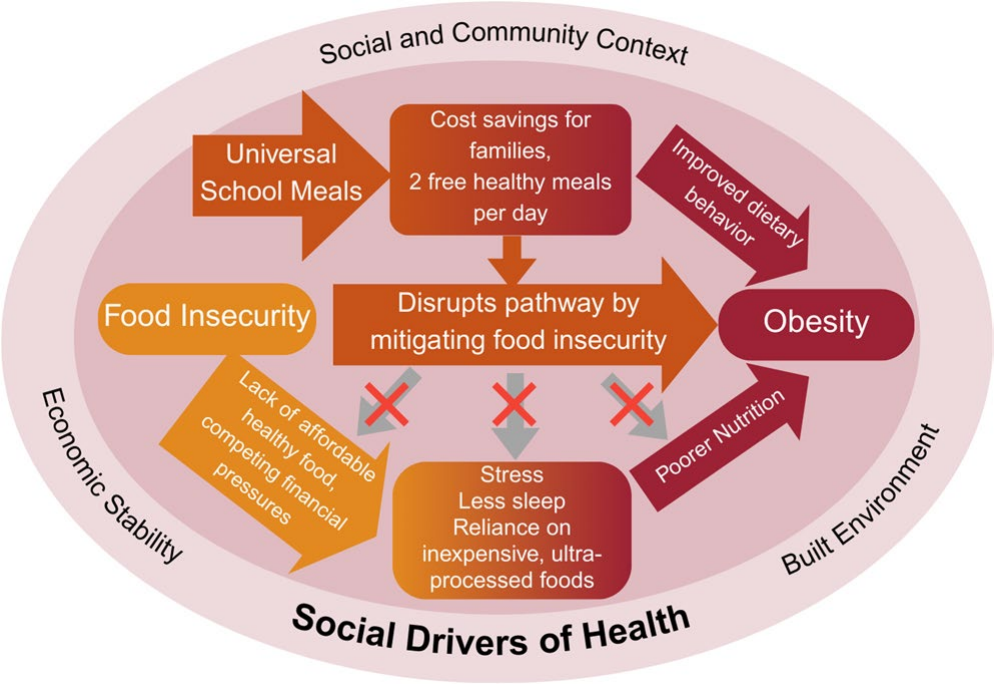
Conceptual Model of USM for Reducing Food Security and Obesity Risk

We propose a conceptual model to illustrate the intersections between obesity prevention, food security, and equity, and the role that USM can play in disrupting these relationships (see Fig. 1). Multiple social determinants of health such as economic stability, neighborhood and built environment, and social and community context act as drivers of the relationship between food insecurity and risk for obesity [3, 8–10]. For example, living in a low-income household limits the ability for families to purchase sufficient food which may increase reliance on less healthy and lower cost options. Living in a neighborhood without a comprehensive grocery store or fresh food options reduces one’s opportunity to purchase whole grains, fruits, and vegetables given proximity to unhealthier, convenient, and ultra-processed items found in corner stores, which are predominantly in low-income and racially minoritized neighborhoods [11]. Thus, the interaction between built environment and social and community context—including historical discrimination and structural racism —exacerbate diet-related disparities through limited public transport and access to fresh, healthy, and affordable foods [12].

Additionally, the role of stress is a key catalyst of the food insecurity-obesity relationship. Greater stress levels result from not being able to afford sufficient food, which can also result in “downgrading” nutrition quality when trying to feed a family. Chronically elevated stress levels have additional potential health implications including poorer sleep quality, which in turn can also impact the types and quantity of foods consumed [3, 9]. Collectively, these determinants explain a substantial amount of the risk for both food insecurity and obesity, especially among low-income and marginalized communities.

Given the focus of USM, which provides free, nutritious meals in schools serving low-income populations, this policy serves to disrupt the relationship between the social determinants of health, food insecurity, and risk for obesity. As highlighted in Fig. 1, UFSM can have a potential positive impact on food security and risk for overweight and obesity through provision of two balanced meals per day to students while also promoting household financial stability and thus food security through cost savings to families. This model highlights several primary mechanisms by which USM policies may reduce the risk of obesity and food insecurity.

### Overview of School Meal Programs

Globally, school feeding programs are one of the most extensive social safety net programs, with over 80% of countries providing school meals to approximately 418 million children daily [13]. In the United States, roughly 30 million children receive a school meal daily through the NSLP, with the majority coming from low-income households and receiving free or reduced priced meals [14]. Importantly, following increased attention on school meals throughout the COVID-19 pandemic and how they are implemented [15–17], there has been a growing movement over the past decade to expand students’ access to free school meals through USM policies [18].

One of the most important provisions to expand access to USM—the Community Eligibility Provision (CEP)— was implemented as part of the Healthy, Hunger-Free Kids Act (HHFKA) of 2010 [19]. CEP enables higher poverty schools and/or districts to provide free breakfast and lunch if at least 25% of students within the school/district are from low-income households and already enrolled in other mean-tested programs—such as the Supplemental Nutrition Assistance Program (SNAP) [6]. Currently, an estimated 27.2 million children attend a school participating in CEP and therefore have access to free school meals through this USM policy [20].

The US also implemented a temporary nationwide USM policy during the COVID pandemic, in part to address the rising rates of food insecurity [16, 21, 22]. Although this national policy ended in June 2022, nine states have subsequently passed state-level USM policies to ensure students continue to have a reliable source of nutritious foods in schools [7]. As a result roughly 60% of schools in the US that participation in the NSLP now provide free meals to all students through CEP and/or state-level USM policies [20].

### School Meals and Childhood Obesity Prevention

Although USM was initially created to streamline meal service and widen the safety net, it is now an evidence-based policy to potentially mitigate obesity risk [23, 24]. This may be through multiple factors along the causal pathway related to the risk of obesity, including through the nutritional quality of the school meals and greater meal participation rates, as well as indirectly through improvements to household finances and food security.

First, school meals are on average one of the healthiest sources of food for children in the US and are on average heathier than those packed from home [25–27]. This is in part due to strong nutrition standards, which were updated as part of the Healthy, Hunger-Free Kids Act of 2010 to align with the concurrent Dietary Guidelines for Americans [19]. As part of these standards, schools are required to provide whole grains, fruits, and a variety of vegetables, as well as limit saturated fats and sodium, and balance minimum and maximum calorie levels. In fact, prior research has found that school meal participation—in the presence of the strong school meal standards in the US—has reduced the risk of obesity among children living in poverty [28]. More recently, the standards were updated again and now place limits on added sugars, as well as further reduce the sodium levels in school meals [29].

Second, there is a substantial body of research documenting the positive impact that USM policies have on school meal participation. Recent systematic reviews have found consistent evidence that CEP and state-level USM policies are associated with greater school meal participation, including among students previously eligible for free school meals—highlighting that stigma may be a key barrier to participation among children from low-income households [30]. While these reviews were conducted prior to state-level USM policies in the US, a recent comprehensive evaluation of the temporary national USM policy and subsequent implementation of state-level USM legislation, as well as CEP implementation, also found substantial increases in school breakfast and lunch participation [31].

There is also a small but growing body of research examining direct associations between USM and BMI. In a recent international systematic review, several studies found a potentially protective effect of USM on BMI [23]. Recent evidence from a state-wide longitudinal study in California demonstrated that schools participating in USM were associated with a 0.60-percentage-point decrease in obesity prevalence after policy adoption (95% confidence interval: −1.07 to − 0.14% points, *P* =.01) compared with eligible, nonparticipating schools. This equated to a 2.4% relative reduction in obesity when accounting for baseline prevalence [24]. These data highlight the impactful role that USM can have on improving food security and reducing risk for childhood overweight and obesity. However, while research examining implementation of healthier meals under the HHFKA has found reductions in obesity among children from low-income households [28], more research is needed specifically examining USM policies in the US and if it reduces disparities in obesity rates among subpopulations, including those from economically constrained households.

### School Meals and Food Insecurity

USM policies are designed to promote nutrition and food security directly by providing free meals to all students and may have indirect benefits as well by offsetting household expenses. Prior systematic reviews have documented the evidence for USM as a tool to reduce food insecurity [23, 32] with two studies demonstrating a positive impact on food security [33, 34]. While these studies were conducted prior to the significant shift toward USM policies more recent, larger scale studies have further shown the positive impact of adoption of USM on food insecurity; one study found that statewide USM adoption was associated with a 12% lower prevalence in food insecurity compared to states without USM adoption and implementation [35]. Additionally, a study conducted in 2022 to examine the relationship between CEP exposure and household spending on groceries found a 19% decrease in spending following adoption of CEP in catchment school districts [36]. These data highlight the impact of USM on reducing hunger and freeing up financial resources within households and emphasizes a “double benefit” on physical and financial health for families at risk for food insecurity and obesity.

Despite the positive associations between USM, BMI, and food insecurity, there is a lack of understanding on how USM adoption mitigates the disparities in obesity and food insecurity that particularly places low-income and racial and ethnic minoritized populations at a disadvantage [3, 4]. Because data are often collected and analyzed at the school or district level, this may mask the potentially greater impact on students within schools who are most at-risk for food insecurity and obesity, such as those living in low-income settings and who are socially minoritized due to their racial or ethnic identity. Therefore, more refined analyses at the student level are needed to determine if these policies are reducing current disparities in obesity and food security.

Further, to improve our understanding of how USM implementation can elicit equitable benefits with a particular focus on marginalized populations, innovative and community-engaged methods are needed. Such work should center the voices of program implementers (i.e., food service, teachers, and administrators) and recipients (students and parents) to develop new ways to implement USM to maximize benefits and further reduce disparities. For example, researchers are currently applying innovative implementation mapping methods [37, 38] in collaboration with the School District of Philadelphia – one of the nation’s poorest cities – to develop and test strategies to improve implementation and participation in school meals. This work centers on implementation science and health equity frameworks [39, 40] to understand key determinants to equitable implementation and impact of USM and to guide the development of equity-focused strategies for implementation; these strategies are currently being tested through a hybrid implementation-effectiveness trial design. Key outcomes of this work are reach (i.e., participation), cost, and sustainability of implementation, as well as health outcomes including food insecurity and weight status. This work highlights how health equity can be centered in advancing the science of USM and its impact on reducing food insecurity.

### Policy Levers and Recommendations

While increasing access to healthy school meals through USM policies is an essential step, additional complimentary policies are needed to maximize the benefits of USM related to obesity and food security. There is already a substantial body of research examining policies to further improve school meal participation and consumption, and these strategies and complimentary policies should be strongly considered [41, 42]. First, policies that ensure sufficient time to eat (e.g., 20 min of mandated seated time at lunch) are essential to ensure students can eat the free meals provided at school. This is particularly important in the context of USM policies, because the increases in participation can also lead to longer lunch lines (and therefore less seated time in the cafeteria to eat). Additional evidence-based approaches include: (1) alternative breakfast models (e.g., breakfast in the classroom, grab-and-go options, or second chance breakfast) to ensure students have access to the free breakfasts served at school; (2) limits on the competitive foods (i.e., snacks and beverages) sold in schools, which typically “compete” with the healthier meals provided; (3) providing more menu choices, including pre-sliced options; (4) enhancing the palatability and cultural appropriateness of the foods offered; and (5) scheduling recess for lunch [41]. Although these strategies have been examined in the context of school meal participation and consumption, further research is needed to examine the associations with student BMI and food security.

## Discussion

School meals represent a holistic intervention that extends beyond nutrition to address multiple determinants of health [18, 20, 23, 35, 43]. This review highlights their potential to reduce both childhood obesity and food insecurity while advancing equity. USM policies—in combination with strong nutrition standards—improve dietary quality and participation, particularly among low-income students. Further, evidence suggests these programs can mitigate obesity risk for children [44] and alleviate household financial strain [35, 36], underscoring their dual benefit for physical and economic well-being.

However, current research has limitations. Most studies are observational, with few longitudinal or randomized designs, and there is an insufficient understanding of how USM impacts disparities across racial and socioeconomic groups [23]. Additionally, implementation challenges—such as time constraints and cultural food preferences—may hinder effectiveness [37]. Future research should prioritize equity-focused implementation strategies and examine long-term outcomes, including health trajectories and potential for health care savings.

The findings from this review also have potential policy implications. First, policymakers at the national level can consider expanding USM nationwide (and/or greater reimbursement rates for schools participating in CEP to maximize participation), as well as standards for adequate lunch periods. Similar policies can also be considered at the state level, including state-wide USM policies. School districts can also consider adopting complementary measures such as adequate lunch periods and culturally responsive menus [45]. Lastly, as federal standards evolve to limit added sugars and ultra-processed foods, school meals may further their impact on obesity prevention and food equity in combination with USM policies.

## Conclusion

School meal programs, particularly Universal School Meals (USM), represent a powerful yet underutilized strategy to address childhood obesity and food insecurity while advancing health equity. Evidence demonstrates that USM improves diet quality, reduces obesity risk, and alleviates household financial strain. To maximize these benefits, policymakers should prioritize sustained funding for USM and expand access through mechanisms like CEP. Complementary policies—such as ensuring adequate time for meals, limiting competitive foods, and enhancing cultural relevance of meals—are essential to optimize participation and consumption. At the district level, schools should adopt innovative breakfast models, improve menu appeal, and engage families and communities in implementation strategies. Future research should focus on equity-driven approaches and evaluations to ensure that USM policies reduce disparities among low-income and racially minoritized populations. Investing in USM is not only a nutritional intervention but a social policy that has strong potential to improve health outcomes in children and promote economic stability. 

## Key References

- Localio AM, Knox MA, Basu A, Lindman T, Walkinshaw LP, Jones-Smith JC. Universal Free School Meals Policy and Childhood Obesity. Pediatrics. 2024. doi: 10.1542/peds.2023-063749.
  - ◦ Universal school meals are associated with reduced risk of childhood obesity.
- Piekarz-Porter E, Cohen J, Schermbeck RM, Leider J, Agurs-Collins T, Chriqui JF. State Laws Leveraging the Community Eligibility Provision to Build Healthy School Meals for All: A Content Analysis. Journal of the Academy of Nutrition and Dietetics. 2025;125(10):1583-91.e5. doi: https://doi.org/10.1016/j.jand.2025.02.006.
  - ◦ Policies in 30 states are promoting community eligibility provision to support universal school meals.
- Spill MK, Trivedi R, Thoerig RC, Balalian AA, Schwartz MB, Gundersen C, et al. Universal Free School Meals and School and Student Outcomes: A Systematic Review. JAMA Network Open. 2024;7(8):e2424082-e. doi: 10.1001/jamanetworkopen.2024.24082.
  - ◦ Universal school means improve students’ dietary behavior and academic achievement.
- McLoughlin GM, Kerstetter M, Yohannes Y, Martinez O, Jones RM, Brownson RC, et al. Understanding implementation determinants of universal school meals through an equity-driven mixed methods approach. Implementation Science Communications. 2025;6(1):44. doi: 10.1186/s43058-025-00713-0.
  - ◦ Numerous implementation challenges to implementing universal school meals exist.
- Cohen JFW, Stowers KC, Odoms-Young A, Franckle RL. A Call for Theory to Guide Equity-Focused Federal Child Nutrition Program Policy Responses and Recovery Efforts in Times of Public Health Crisis. J Acad Nutr Diet. 2023;123(1):15-28. doi: 10.1016/j.jand.2022.07.016.
  - ◦ Equity-informed implementation of universal school meals can enhance their impact.

## Data Availability

No datasets were generated or analyzed during the current study.
